# Criticality of Surface Characteristics of Intravenous Iron–Carbohydrate Nanoparticle Complexes: Implications for Pharmacokinetics and Pharmacodynamics

**DOI:** 10.3390/ijms23042140

**Published:** 2022-02-15

**Authors:** Felix Funk, Beat Flühmann, Amy E. Barton

**Affiliations:** Vifor Pharma Management Ltd., Flughofstrasse 61, CH-8152 Glattbrugg, Switzerland; felix.funk@viforpharma.com (F.F.); beat.fluehmann@viforpharma.com (B.F.)

**Keywords:** nanomedicine, iron–carbohydrate complexes, carbohydrate

## Abstract

Un-complexed polynuclear ferric oxyhydroxide cannot be administered safely or effectively to patients. When polynuclear iron cores are formed with carbohydrates of various structures, stable complexes with surface carbohydrates driven by multiple interacting sites and forces are formed. These complexes deliver iron in a usable form to the body while avoiding the serious adverse effects of un-complexed forms of iron, such as polynuclear ferric oxyhydroxide. The rate and extent of plasma clearance and tissue biodistribution is variable among the commercially available iron–carbohydrate complexes and is driven principally by the surface characteristics of the complexes which dictate macrophage opsonization. The surface chemistry differences between the iron–carbohydrate complexes results in significant differences in in vivo pharmacokinetic and pharmacodynamic profiles as well as adverse event profiles, demonstrating that the entire iron–carbohydrate complex furnishes the pharmacologic action for these complex products. Currently available physicochemical characterization methods have limitations in biorelevant matrices resulting in challenges in defining critical quality attributes for surface characteristics for this class of complex nanomedicines.

## 1. Introduction

Iron deficiency is a global health problem that is associated with a wide breadth of underlying conditions including chronic kidney disease, heart failure, underlying inflammatory conditions, cancer, and bariatric surgery, as well as menorrhagia and post-partum bleeding [1]. Iron is not only the key driver for erythropoiesis and formation of red blood cells [2]. Iron is also a critical element in cellular processes including mitochondrial energy metabolism, and iron also catalyzes many fundamental enzymatic reactions in various tissues. Thus, the effects of iron deficiency clinically are broad across organ systems and include impaired function of the myocardium and skeletal muscle, immune system effects, and cognitive and neuronal development. Many of the diseases associated with iron deficiency are chronic and require repeated doses of intravenous iron–carbohydrate complexes due to the limited efficacy and tolerability of oral iron salts [3]. Many oral ferrous iron salts (e.g., sulfate and fumarate) have been formulated as potential iron deficiency anemia (IDA) treatments; however, as scientific knowledge about anemia in hematology developed, it became evident that not all iron-deficient patients responded to oral iron salts. Intravenous iron products were initially developed to address poor oral absorption and gastrointestinal side effects associated with oral iron salt treatments. Early studies attempted to inject iron salts or un-complexed polynuclear ferric oxyhydroxide but patients experienced serious adverse events related to the very rapid dissolution of iron after injection [4,5]. In 1932, Heath and colleagues parenterally administered doses of iron ammonium citrate ranging from 8–32 mg to 17 patients. Subjects were also given oral iron after conclusion of the parenteral iron therapy. Parenteral iron was not as effective in increasing hemoglobin compared to oral iron. The authors stated, “it is therefore evident that there is no distinct advantage to giving iron parenterally”. Parenteral administration of iron in these formulations was also associated with “severe and dangerous” side effects including hypotension, tachycardia, nausea, and vomiting. The authors concluded that “… except in rare instances, the administration of iron by the parenteral route should be avoided”. In a subsequent study by Goetsch et al. in 1946, eight patients received either colloidal ferric hydroxide or ferric oxide intravenously [5]. Reactions noted were described as “severe and often alarming” and included facial flushing, swelling and stiffness of the tongue and face, vomiting and hypotension, which necessitated terminating the infusion in three patients. The authors concluded that “there can be no doubt that the reaction to iron parenterally administered in large doses are great enough to contraindicate use of this measure as a therapeutic procedure.”

Thus, simple iron salts and “uncoated” iron oxide administered parenterally do not appear to efficiently reach the bone marrow and likely rapidly distribute into other tissues (e.g., endothelial cells) and induce serious adverse reactions [6]. These early attempts to administer iron parenterally were clearly illustrative of the need for better designed active ingredients to enable safe and effective iron supplementation.

Iron–carbohydrate complexes were developed to address the severe toxicity issues associated with earlier iron supplementation products. Carefully designed carbohydrate ligands were complexed and bonded to polynuclear iron cores to furnish the pharmacologic activity safely and effectively. The ideal active ingredient for an intravenous iron–carbohydrate complex should be designed in a manner so as not to release iron too quickly to avoid adverse events such as those documented after ferric hydroxide administration [5]. In addition, the size of the entire complex inclusive of carbohydrate content is designed to avoid anaphylactic reactions [7,8]. The iron–carbohydrate complexes are intended to facilitate clearance from the serum by macrophages and delivery to the liver and spleen to provide iron to the physiological iron storage and transport system. 

It is important to note that contemporary intravenous (IV) iron–carbohydrate complexes are designed to be similar to endogenous serum ferritin, which safely stores iron within a protein and releases iron based on homeostatic demand [7,9]. They all consist of polynuclear iron-oxyhydroxide cores, which are complexed by their respective carbohydrates and can therefore be described as polymer complexes or nanoparticle complexes [10]. This illustrates the criticality of the carbohydrate component of the active ingredients for commercially available IV iron–carbohydrate complexes. As discussed in more detail below, these widely used iron–carbohydrate complexes have significantly different physicochemical properties, including parameters related to size and molecular weight, structure, surface properties, and reactivity. They are differentiated by their kinetic profiles and by their thermodynamic properties, which are inextricably linked to the entire iron–carbohydrate complex. Additionally, their nanoparticle surface characteristics and polydispersity profiles, which are also dictated by the bonded carbohydrates, drive the rate and extent of uptake into the monocyte phagocytic system and the rate and extent of biodegradation [11]. Thus, by design, the chemistry of the bonded carbohydrate is fundamental in furnishing the pharmacologic action for this class of drugs.

The development of intravenous iron–carbohydrate nanoparticles to treat iron deficiency has evolved significantly over the past 70 years. Intentional design of the carbohydrate has been the predominant focus to innovate and optimize the surface characteristics of the active ingredient complex. The carbohydrate characteristics functionalize the surface and fundamentally drive clearance from plasma, cell uptake, and biodegradation. The types of carbohydrates used in this capacity are highly heterogeneous (e.g., dextrans, sucrose, and carboxymaltose), and it is the complex of the carbohydrate portion in conjunction with how it is bound to the iron core that defines the overall surface characteristics, particle size, particle size distribution, and particle morphology. Due to the complexity of these nanoparticle formulations, manufacturing conditions, processes and controls are critical to limiting batch to batch variation and to ensure product quality and therefore consistent in vivo performance [12]. The surface characteristics are also fundamental in furnishing pharmacologic activity, plasma pharmacokinetic and biodistribution profiles, and adverse events profiles including hypersensitivity reactions. In summary, surface carbohydrate components of intravenous iron nanoparticle preparations do not just function as stabilizing agents but rather drive their behavior in vivo and, therefore, the entire nanoparticle complex defines the active pharmaceutical ingredient. This review will cover the various types of carbohydrate coatings, their implications on in vivo behaviors, and relevant challenges in characterizing this portion of the nanoparticle preparation.

## 2. Different Carbohydrate Types Are Used as the Ligands of the Polynuclear Iron Cores

In the approximately 90 years of history of parenteral iron administration, there have been a multitude of preparations developed, and several are still used in clinical practice [13]. Many of the early preparations were withdrawn from the market due to adverse reactions and difficulty with manufacturing procedures. Subsequently, second and third generations of preparations were developed based on experiences with the advantages and disadvantages of first generation preparations. However, one very old preparation, iron sucrose, developed over 70 years ago, is still successfully on the market, highlighting the careful development at that time, in particular to mitigate anaphylactic reactions. 

### 2.1. The Correct Conformation of the Iron Carbohydrate Complex Is Required to Produce a Safe Iron–Carbohydrate Complex

Despite the relative perceived simplicity of manufacturing iron–carbohydrate complexes, it has been clearly demonstrated that the manufacturing process at all check points is critical to ensuring safe and effective therapeutic agents [12]. Even slight changes in alkali content, sucrose content, carbonate content, and pH of precipitation point can dramatically affect safety profiles of the finished drug product. This was illustrated when Nissim compared intravenous infusions of solutions of colloidal ferric hydroxide and saccharated iron oxide [14]. Four patients receiving ferric hydroxide developed definite pyrexial reactions, three of them rather severe. However, out of twenty-five administrations of 100 mg or more of saccharated iron oxide, only four were followed by reactions, and the association of the iron preparation with two of the reactions was deemed unclear by the authors. 

Subsequently, in 1949, Nissim and Robson systematically investigated manufacturing parameters to obtain saccharated iron oxide in a standardized way in order to prevent different toxicities in different batches, as observed previously [14,15]. The parameters varied included alkali content, sucrose content, carbonate content, and pH of the precipitation point. The batches were tested for toxicity in mice. The survival of animals differed between batches containing different amounts of sucrose but otherwise identical manufacturing conditions. Survival was lowest at 10 days (0%) with the highest g/g sucrose content, and survival was highest (90%) with the lowest g/g sucrose content. This demonstrates the importance of the optimal balance of both the type of ligand as well as the amount of ligand. A similar trend was observed regarding pH of precipitation points with preparations with higher pH points showing lower survival rates compared to lower pH points (3.7–4.7). Collectively, these data demonstrate that iron–carbohydrate complexes can produce much different safety profiles when the concentration of the carbohydrate ligand or the manufacturing process is manipulated. Thus, it is clear that both the polynuclear iron-oxyhydroxide and the sucrose as an entity, i.e., as the iron oxyhydroxide carbohydrate complex, are fundamental and do not simply provide processing functions. Clearly, the early studies by Heath et al. and Goetsch et al. [4,5] suggested that neither low-molecular iron complexes nor naked iron (oxy)hydroxides preparations were suitable for parenteral administration. In contrast, with the saccharated iron oxide complex which showed a much better safety profile, Nissim et al. also clearly demonstrated the importance of a standardized manufacturing process for this complex. 

In the following decades, a large number of polynuclear iron oxyhydroxide complexes were developed, with the primary aim to improve safety and efficacy profiles as well as increase the rate and dose of administration [7]. This was achieved through innovation focused on the surface characteristics produced by the carbohydrate ligand. In Table 1, the various types of ligands are presented with examples of previous and currently marketed drugs. They are polynuclear complexes analogous to iron tightly bound within ferritin, with the protein ligand replaced by a carbohydrate component because, given by the parenteral route, ferritin has antigenic properties [16]. In order to avoid potential anaphylactic reactions, only carbohydrates and no proteins have been used for parenteral iron preparations.

### 2.2. The Carbohydrate Ligands of the Iron–Carbohydrate Preparations Are Complex and Structurally Heterogeneous

There are six main iron–carbohydrate preparations currently on the market: iron sucrose (Venofer^®^), sodium ferric gluconate (Ferrlecit^®^), iron dextran (Cosmofer^®^), ferric derisomaltose (Monofer^®^), ferric carboxymaltose (Ferinject^®^), and ferumoxytol (Feraheme^®^). Their composition is presented in Table 2. Iron sucrose contains the disaccharide sucrose as ligand, i.e., a low-molecular-weight carbohydrate. The drug product contains 20 mg elemental iron/mL and approximately 30% sucrose w/v (300 mg/mL), i.e., 15 mg sucrose/mg iron. The drug product has a pH of 10.5 to 11.1. The proposed structural formula is [Na_2_Fe_5_O_8_(OH)⋅3(H_2_O)]_n_⋅m(C_12_H_22_O_11_) [17]. 

Sodium ferric gluconate is the sodium salt of a ferric ion carbohydrate complex in an alkaline aqueous solution with 12.5 mg iron/mL and approximately 20% sucrose w/v, i.e., 16 mg sucrose/mg iron. In contrast to iron sucrose, the drug product has a pH 7.7–9.7. The structural formula is considered to be [NaFe_2_O_3_(C_6_H_11_O_7_)(C_12_H_22_O_11_)_5_]_n≈200_ [18]. According to this formula, it contains one gluconate, also a low-molecular-weight ligand per two iron or 1.7 mg gluconate/mg iron. 

Low-molecular-weight iron dextran contains 50 mg iron/mL. The pH of the solution is between 4.5 to 7.0 [19]. The content of iron(III)-hydroxide dextran complex is 312.5 mg/mL [20]. From this content determination, it can be calculated that the dextran content is approximately 206 mg/mL or 4.1 mg dextran/mg iron.

Ferric derisomaltose, also referred to as iron isomaltoside 1000, contains 100 mg iron/mL. Isomaltoside 1000 consists predominantly of 3–5 glucose units and originates from a chemical modification of isomalto-oligosaccharides present in Dextran 1 (European Pharmacopoiea) [21]. 

Ferric derisomaltose has the following empirical formula: {FeO^(1−3X)^ (OH^)(1+3X^) (C_6_H_5_O_7_ ^3−^)}_X_, (H_2_0)_T_, (C_6_H_10_O_6_)_R_(-C_6_H_10_O_5_^-^)_Z_(C_6_H_13_O_5_)_R_, (NaCl)_Y_; X = 0.0311; T = 0.25; R = 0.14; Z = 0.49; Y = 0.14 [21,22]. From this formula, it can be derived that the complex contains approximately 2.3 mg derisomaltose/mg iron. As the formula further shows (and can also be seen from the name in the former German prescribing information [23]), iron citrate isomaltoligosaccharide alcohol–hydrate complex, ferric derisomaltose also contains citrate as an additional ligand in a concentration of approximately 10 mg/mL or 0.1 mg citrate/mg iron. The drug product is a solution with pH 5.0–7.0.

Ferric carboxymaltose contains 50 mg iron/mL. The complex has the following empirical formula: [FeO_x_(OH)_y_(H_2_O)_z_]_n_ [{(C_6_H_10_O_5_)_m_ (C_6_H_12_O_7_)}_l_]_k_, where n ≈ 10^3^, m ≈ 8, l ≈ 11, and k ≈ 4 (l represents the mean branching degree of the ligand). The ligand carboxymaltose is obtained from maltodextrin by oxidation [24]. The drug product is a solution with pH 5.0–7.0. The complex contains approximately 75 mg carboxymaltose/mL (approximately 1.5 mg carboxymaltose/mg iron).

Ferumoxytol contains 30 mg of iron/mL. The complex is an iron oxide coated with polyglucose sorbitol carboxymethylether [25]. The chemical formula of ferumoxytol is Fe_5874_O_8752_-C_11719_H_18682_O_9933_Na_414_ [26]. Based on this formula the complex contains approximately 0.97 mg carbohydrate/mg iron. Polyglucose sorbitol carboxymethylether was identified as a dextran with low degree of branching (1–2%), partly carboxymethylated at positions C-2, C-3, or C-4 in the glucan backbone and with a reduced, non-carboxymethylated C-1 chain end unit [25]. The ferumoxytol drug product is formulated with mannitol (1.5 mg mannitol/mg iron). It has a pH of 6 to 8 [26].

### 2.3. The Bond Interactions between the Polynuclear Iron Core and Carbohydrate Ligands Differ among the Iron–Carbohydrate Preparations

There are multiple bond energies within and between molecules that may include covalent bonds, coordination bonds, ionic–ionic interactions, ionic–dipole interactions, e.g., hydrogen bonds, and Van der Waals forces. In addition, effects such as chelation or steric hindrance contribute to the stability of an interaction. All these bonds have different strengths, as the following examples show. The strengths of most hydrogen bonds lie between 10 and 40 kJ mol^−1^ or approximately 5–10 kT per bond at 298 K, which makes them stronger than a typical van der Waals bond (~1 kJ mol^−1^ or ~1 kT) but still weaker than covalent or ionic bonds (~500 kJ mol^−1^ or ~100 kT) [31]. kT is the thermal energy, where k is the Boltzmann constant (1.3807 * 10^−23^ J/K) and T is the temperature in Kelvin.

The same forces as for small low-molecular-weight molecules or complexes are also responsible for the interactions between the surfaces of particles, but they can manifest themselves in quite different ways and lead qualitatively to new features when acting between large particles or extended surfaces [31]. There is a difference in assessing bond energies between more simply structured, small low-molecular-weight molecules and more complex high-molecular-weight iron–carbohydrate complexes. For small, low-molecular-weight molecules, there are a low number of interactions, and these only consist of hydrogen bonds or Van der Waals forces, and the overall interaction is not strong. However, in complex high-molecular-weight iron–carbohydrate complexes, the iron core undergoes many interactions with the ligands, which effectively prevents it from interacting with other iron cores. In order to obtain a sufficiently high stability, the different intravenous iron–carbohydrate preparations reflect slightly different approaches with regard to the interacting forces, but they are substantially driven by hydrogen bonds. Obviously, the contribution of the different forces cannot be quantitatively determined, but it is interesting to see how the lack of minor quantities of certain forces is compensated by others. 

For example, in the case of iron sucrose, the ligand has a low molecular weight. The ligand amount compared to the iron content as well as its molar concentration are therefore quite high. In addition, the pH in the final diluted preparation is high (≥10.5), enabling an increased formation of hydrogen bonds between deprotonated hydroxy groups of the ferric oxyhydroxide core and the hydroxy groups of the sucrose. For sodium ferric gluconate, the absolute sucrose concentration is lower than in the iron sucrose preparation, but the amount compared to the iron content is similar and the pH is also lower. However, there is a compensatory factor due to the presence of gluconate, which forms a coordinative bond and even a chelate with the ferric oxyhydroxide [32]. Its chelation to iron can also drive the deprotonation of nearby hydroxyl groups, further enhancing the complex stability [33]. Iron dextrans clearly have ligands with a much higher molecular weight compared to iron sucrose and sodium ferric gluconate. This allows the formation of multiple hydrogen bonds between the ferric oxyhydroxide and the dextran. Although the absolute amount of carbohydrate is similar to that in sodium ferric gluconate complex, the amount relative to the iron content is much lower. For iron isomaltoside, there is a similar absolute amount of ligand present as on iron dextran, and the amount relative to the iron is even lower. In addition, there is a small amount of citrate present, a ligand that can form chelate complexes with iron. Ferric carboxymaltose contains carboxymaltose, a ligand unifying the potential of multiple hydrogen bonds and a coordinative bond within the same molecule, the carboxylate forming a coordinative bond and a chelate with adjacent hydroxy groups. The absolute and relative amounts of the carbohydrate are lower than for all of the preparations described above. The same holds true for the ligand of ferumoxytol, in which in addition to the hydrogen bonds, the carboxymethylated species can also form the coordinative bonds. Interestingly, ferumoxytol also contains maltose as an excipient, which could also contribute to the stability, but due to the relatively low concentration and the almost neutral pH of the solution, this effect is considered to be minor. An overview of bonds is given in Table 3.

All preparations have been documented to be stable in colloidal suspension, as described below. Shah et al. reported their physicochemical characterization and thermodynamic stability assessment for the colloidal iron drug product iron sucrose [34]. The complex deformed at low pH and reformed back at the formulation pH. The complex was stable under mild-to-moderate temperature <50 °C. The resistance of the complex to breakdown by electrolytic conditions, excipient dilution, ultracentrifugation, and the reversible complexation after alteration of formulation pH suggest iron sucrose is a lyophilic colloid in nature and lyophilic colloids are thermodynamically stable. The authors concluded that “all relevant components play a direct role in the “in situ” generation of this equilibrium coordination complex, which constitutes the active ingredient in iron sucrose.” Similarly, Yang et al. conducted a thermodynamic stability assessment of the colloidal iron drug product sodium ferric gluconate [35]. A high-performance gel permeation chromatography (HP-GPC) method was developed, validated, and used to determine the molecular weight (MW) of sodium ferric gluconate following various stress conditions. The MW of sodium ferric gluconate remained unchanged after inducing various stress conditions including basic buffer dilution (pH of 8 and 9) and ultracentrifugation. However, sodium ferric gluconate showed signs of instability at higher temperatures (>90 °C) after 30 days and at a pH of 10–11. Differences in stability profiles under dilution conditions have been demonstrated with the reference listed iron sucrose complex compared to follow-on copies of iron sucrose complexes (e.g., iron sucrose similars) [36]. Various dilutions of samples of iron sucrose and iron sucrose similars (approved follow-on copies) were investigated by dynamic light scattering (DLS) to evaluate changes in particle size distribution. Statistically significant differences in particle size and distribution were observed between the reference listed drug product and the iron sucrose similars despite meeting sameness parameters outlined by the regulatory agencies approving the iron sucrose similars. Philipp et al. assessed the physicochemical stability of colloidal ferric carboxymaltose when diluted and stored in polypropylene (PP) bottles and bags for infusion [37]. Samples were diluted (500, 200, and 100 mg iron in 100 mL saline) and stored at 30 °C and 75% ± 5% relative humidity (rH) for 72 h, and samples were withdrawn aseptically at preparation and after 24, 48, and 72 h. Multiple parameters were used to test stability-related measures (pH, total iron, and iron(II) content, molecular weight range determination, microbial contamination and particles count ≥ 10 μm). Under the tested experimental conditions, colloidal ferric carboxymaltose solution diluted in saline in PP infusion bottles or bags demonstrated physical and chemical stability for up to 72 h at 30 °C and 75% rH. There have not been any published studies of the thermodynamic stability of iron dextran complexes. According to the prescribing information of iron dextran, the complex is so stable that it only splits after uptake into the reticuloendothelial system [19]. Similarly, in the Swiss prescribing information of iron isomaltoside 1000, in the pharmacokinetics section, it is stated (translated) [38] that “after intravenous administration the iron(III)-isomaltoside 1000 is rapidly taken up by the reticuloendothelial system (RES), particularly in the liver and spleen. From there the iron is slowly released”. A similar statement in the United States prescribing information describes the stability of ferumoxytol: “Ferumoxytol is the only ferromagnetic iron–carbohydrate complex available for clinical use in iron deficiency. It consists of a superparamagnetic iron oxide that is coated with a carbohydrate shell, which helps to isolate the bioactive iron from plasma components until the iron–carbohydrate complex enters the reticuloendothelial system macrophages of the liver, spleen and bone marrow. The iron is released from the iron–carbohydrate complex within vesicles in the macrophages” [26].

## 3. The IV Iron–Carbohydrate Complexes Have Significantly Different Physicochemical Properties

Due to the different carbohydrates used and the different manufacturing conditions, the iron–carbohydrate complexes have significantly different physicochemical properties. These include parameters related to size and molecular weight, structure, surface properties, and reactivity, which might be relevant for the pharmacological effects in the body. Some of the physico-chemical properties that have been identified as essential are summarized in Table 4.

## 4. The IV Iron–Carbohydrate Complexes Have Significantly Different Pharmacological Effects in the Body, Indicating the Importance of Surface Characteristics

Numerous non-clinical and clinical studies have been conducted comparing different iron–carbohydrate complexes. Differences depending on the different iron–carbohydrate complexes were observed in (a) the clearance kinetics from the serum, (b) tissue distribution, (c) the pharmacodynamics, and (d) the safety profiles. This clearly indicates that the specific iron–carbohydrate complexes are responsible for furnishing the pharmacological activity and thus qualify for active ingredients.

### 4.1. The Pharmacokinetics of Iron–Carbohydrate Complexes Are Different and the Rate and Extent of Iron Exposure Is Inextricably Linked to the Ligand

As depicted in Table 5 below, the known pharmacokinetic parameters differ widely amongst even the more similarly configured iron–carbohydrate preparations, iron sucrose and iron gluconate, thus underscoring that even small changes in iron–carbohydrate complexes produce clinically relevant changes in the pharmacokinetic profiles.

### 4.2. Biodistribution Profiles in Key Pharmacologic Target Tissues Differs Widely between Iron–Carbohydrate Complexes 

**Figure 1:**
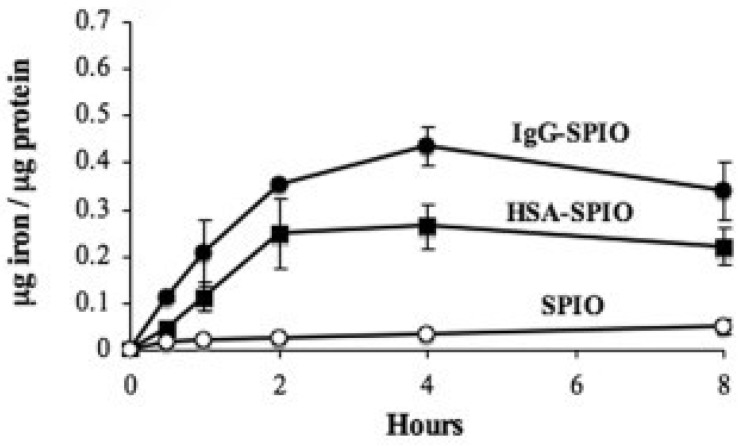
Iron oxide nanoparticle uptake by monocytes and macrophages with different ligands; IgG, human serum albumin and no ligand. SPIO = superparamagnetic iron oxide, IgG = immunoglobulin G, HSA = human serum albumin [45].

Various parameters influence iron oxide nanoparticles biodistribution such as nanoparticle size, hydrophobicity/hydrophilicity, surface charge, core composition, coating properties, route of administration, quantity administered, and opsonization [11]. The ligand bound to the polynuclear ferric oxyhydroxide of the iron–carbohydrate complexes is a principal determinant of the rate and extent of opsonization and subsequent delivery for the pharmacological site of action in the mononuclear phagocytic system [11]. As noted by Dulinska-Litewka et al., “the carbohydrate ligand “can decide about the nanoparticle’s interactions with its biological environment (cells, proteins, etc…)” [44]. The opsonization mechanism has been shown to occur at both the polynuclear iron and ligand surfaces and is influenced by the proteins adsorbed on to the ligand surface which is dictated by the size of the iron–carbohydrate complex [11]. In general, efficiently opsonized iron–carbohydrate complexes have longer half-lives and slower clearance values [11]. Figure 1 demonstrates the clear influence of the bound ligand on the amount of iron uptake by monocytes and macrophages [45].

Once the iron–carbohydrate complex reaches the liver, Kupffer cells biodegrade and metabolize the iron–carbohydrate complex to ferrous iron, which is then stored in ferritin, now ready for transfer via ferroportin to transferrin [11]. There is also evidence that hepatocytes and spleen cells also contribute to the biodegradation of the iron–carbohydrate complex [46]. As noted in the iron dextran prescribing information, “changes in serum ferritin levels represent the changes in calculated cellular non-heme iron levels” [19]. Preclinical studies also indicate that different iron–carbohydrate complexes also impact each product’s biodistribution following administration. Spicher et al. studied several different intravenous iron–carbohydrate complexes and showed that fetal avian tissues can be used to study tissue concentrations in the heart and liver over time [47]. Differences were found between equimolar doses of the iron–carbohydrate complexes (ferric carboxymaltose, sodium ferric gluconate and iron sucrose) and even between a reference product (Venofer^®^) and a follow-on copy [47].

**Figure 2:**
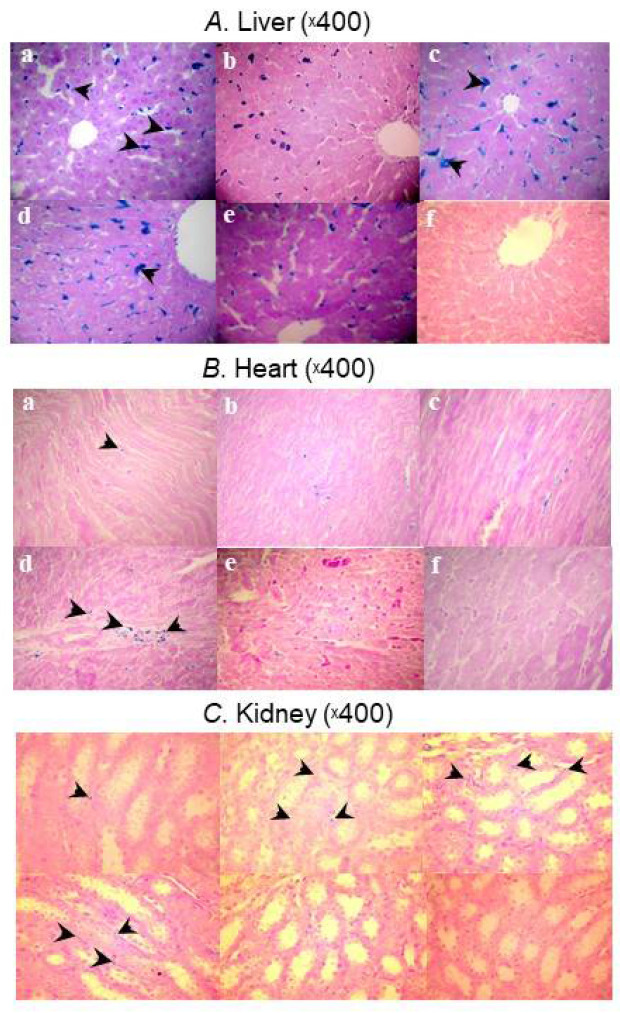
Micrographs showing Prussian blue staining for iron deposits in (**A**) liver, (**B**) heart, and (**C**) kidney samples taken from the HMW iron dextran (**a**), LMW iron dextran (**b**), ferric gluconate (**c**), ferric carboxymaltose (**d**), iron sucrose (**e**), and control (**f**) groups on Day 29 [48].

The biodistribution of five iron–carbohydrate complexes was also investigated in Sprague-Dawley rats [48]. Ten rats (50% male) per group were allocated to receive five doses of 40 mg/kg of ferric carboxymaltose, high-molecular-weight (HMW) iron dextran, low-molecular-weight (LMW) iron dextran, sodium ferric gluconate, or iron sucrose over 4 weeks. Control animals received saline. Prussian blue staining of liver, heart, and kidney tissue showed distinct uptake profiles between the iron–carbohydrate complexes and controls (Figure 2). Livers showed significantly more positive staining for iron in the Kupffer’s cells and hepatocytes of rats treated with HMW and LMW iron dextran and FG compared to rats treated with ferric carboxymaltose, iron sucrose, and isotonic saline solution (*p* < 0.01). In contrast to rats treated with ferric carboxymaltose, iron sucrose, and isotonic saline solution, HMW and LMW iron dextran and sodium ferric gluconate-treated rats showed significant positive staining for iron in the cardiomyocyctes and interstitium of heart tissue (*p* < 0.01). Similar results were observed in the kidneys; rats treated with HMW and LMW iron dextran and sodium ferric gluconate showed a significant positive staining for iron in the tubular epithelial cells as well as in the renal interstitium compared to rats treated with ferric carboxymaltose, iron sucrose, and isotonic saline solution (*p* < 0.01). The authors concluded that differences in the molecular structures and hence stability and reactivity of these compounds may account for the varied safety profiles of the IV iron preparations tested.

### 4.3. The Difference in Pharmacologic Activity between the Different IV Iron–Carbohydrate Complexes Is Further Illustrated by the Pharmacodynamic Marker Ferritin

Ferritin is an optimal pharmacodynamic marker for iron–carbohydrate complexes because it accurately describes how the iron–carbohydrate complex affects the function of the liver and spleen with regard to iron homeostasis. Each iron–carbohydrate complex produces a unique uptake pattern and subsequent positive tissue staining for ferritin, which indicates that the differences in the surface chemistry of the iron–carbohydrate complexes is a driver of the rate and extent of uptake [48]. 

Intravenous iron–carbohydrate complexes are designed for targeted uptake by the resident macrophages of the reticuloendothelial system (RES). After macrophage uptake, the iron–carbohydrate complexes are degraded in the lysosome and a small proportion of iron is transported via ferroportin to transferrin [49]. The intact iron–carbohydrate complex dictates lysosomal uptake and subsequent biodegradation [9]. The majority of iron generated from biodegradation is incorporated into ferritin, where it is tightly bound as ferric iron and safely stored until physiologic homeostatic signaling induces ferroportin expression and requisite amounts of iron are released from ferritin for binding to transferrin. Serum ferritin is considered the most clinically relevant and accessible iron parameter that serves as the primary indicator of stored iron in the RES [50]. However, it should be noted that serum ferritin itself is iron poor compared to tissue ferritin [49]. After injection of intravenous iron–carbohydrate complexes, both total serum iron and transferrin bound iron are only transiently elevated and return to baseline once the nanoparticles are phagocytized by macrophages and incorporated into ferritin. Therefore, both these indices do not accurately reflect the response of the body to the drug (pharmacodynamic profile). Rather, the process of incorporating iron into ferritin accurately reflects the mechanism of storing iron to be over time available for the physiological release to transferrin and finally incorporation into the erythrocyte to increase hemoglobin based on tightly regulated homeostatic mechanisms.

**Figure 3:**
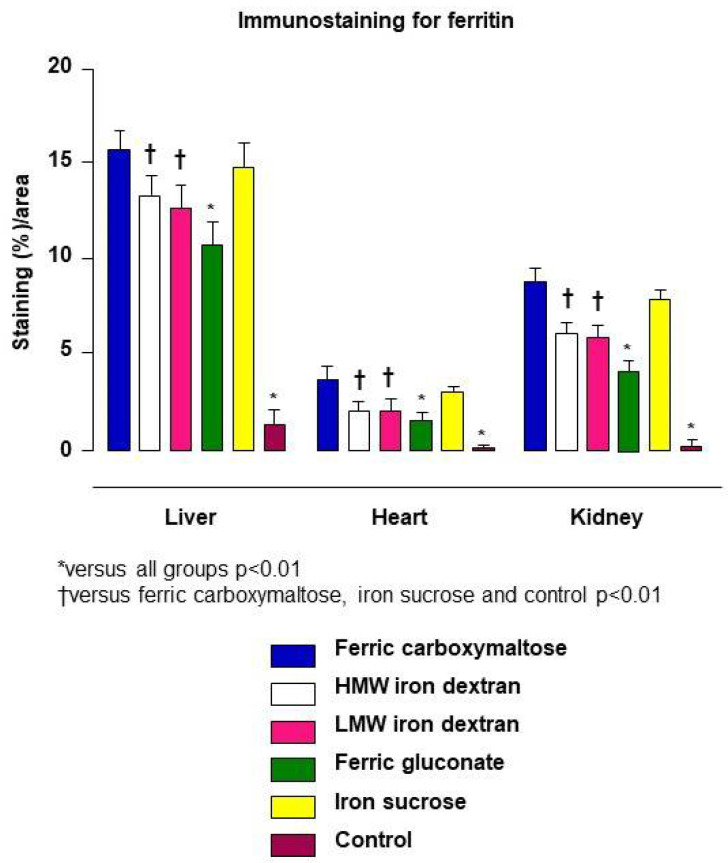
Bar chart showing ferritin immunostaining for stored iron in liver, heart, and kidney samples taken from the HMW iron dextran, LMW iron dextran, ferric gluconate, ferric carboxymaltose, iron sucrose, and control groups on Day 29 [48].

In the previously described study evaluating biodisposition of iron–carbohydrate complexes in rats, immunostaining for ferritin illustrated different profiles in the liver as well as the heart and kidney at a lower magnitude [48]. Compared to control animals, all iron-treated rats showed marked, statistically significant increases on the percent area of the various tissues staining positive for ferritin, indicative of the upregulated ferritin production to safely store iron. Each iron–carbohydrate product produced its own tissue uptake pattern represented by positive staining for ferritin, which indicates that the differences in the structure of the iron–carbohydrate complexes drive uptake (Figure 3). 

**Figure 4:**
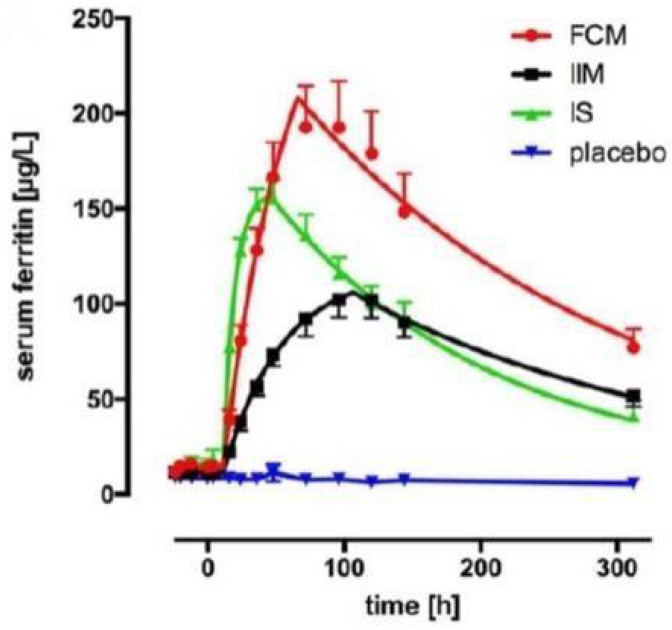
Cumulative serum ferritin profiles for treatment groups, fitted using global fitting with a custom function (plateau followed by mono-exponential association followed by mono-exponential decay), FCM = ferric carboxymaltose, IIM = iron isomaltoside, IS = iron sucrose [43].

Serum ferritin represents the rate and extent of the incorporation of iron derived from iron–carbohydrate complexes into the physiological iron stores and is the most robust endpoint for construction of dose and exposure pharmacokinetic and pharmacodynamic models for this class of injectable complex drugs. A recent study examined the pharmacokinetic and pharmacodynamic profiles of three iron–carbohydrate complexes, ferric carboxymaltose, iron isomaltoside, and iron sucrose [43]. A dose of 200 mg of complexed polynuclear iron was administered to otherwise healthy anemic human subjects (*n* = 8 per arm). Eight subjects received saline (placebo group). Serum samples were collected up to 13 days (312 h) post-administration. Serum ferritin increased following administration of all three iron–carbohydrate complexes compared to placebo, and each iron–carbohydrate produced a unique serum ferritin profile (Figure 4), which translated into statistically significant differences in area under the time–concentration curve for ferritin.

### 4.4. The Surface Characteristics Confer the Relative Immunogenicity Risk of Iron–Carbohydrate Complexes

The risk of immune reactions, specifically anaphylactic type reactions, associated with the various commercially available iron–carbohydrate complexes has been described in a multitude of pharmacovigilance reports over the past several decades [51,52]. These reactions have been attributed to the surface characteristics and interaction with the innate immune system [53] and have necessitated test doses and post-administration observation periods with some preparations [19,26]. An in vitro study evaluated the dextran-associated immunogenicity of several commercially available preparations as well as the isolated carbohydrate ligand of iron isomaltoside 1000 [54]. The study showed that the isomaltoside 1000, the ligand of iron isomaltoside 1000 acted as a hapten, binding and “neutralizing” the antibodies, but not leading to precipitated antibody complexes. In contrast, with the entire iron isomaltoside complex, immune complex formation was observed. The authors hypothesized that the proximity of the individual haptens on the surface of the entire iron–carbohydrate complex is responsible for the observed precipitated antibody complexes. This strongly suggests that the nanoparticles surface structure is contributing to the pharmacological activity. As the nanoparticulate iron–carbohydrate complex has pharmacological properties distinct from a mixture of its isolated components, the entire iron–carbohydrate complex has to be considered as the active pharmaceutical ingredient.

## 5. The Entire Iron–Carbohydrate Complex Defines Properties Relevant to Furnish Pharmacological Activity

As discussed, the composite interactions of the polynuclear core with each intravenous iron preparation’s surface carbohydrate ligands dictates the unique pharmacologic and safety profiles of the commercially available agents. The physicochemical characteristics presumed to be influential on the pharmacokinetic profile of IV iron nanomedicines include: total iron content, iron core crystal structure, type of carbohydrate surface ligand, nanoparticle shape and size, nanoparticle surface characteristics, and type and quantity of plasma proteins adsorbed to the particle (e.g., the protein corona). There are substantial differences in the properties of the different preparations. However, it must be noted that different methodologies and/or experimental conditions might affect the outcome of the physicochemical characterization study. There are many characterization parameters that have been defined as critical quality attributes (CQAs) for iron–carbohydrate complexes but there are also CQAs that have not yet been fully identified and measured for these widely used drug products. Importantly, analytical methods to measure these CQAs are much more complex than for small molecules, and for some CQAs, these methods may not be reproducible and fully validated [12].

The current orthogonal methodology is not sufficient to fully describe the surface structure, which is presumed to be very different for different carbohydrates and relevant for interaction with serum components [55]. Obtaining robust data on nanoparticles’ size, charge, and other physical and chemical properties (e.g., colloidal stability and dissolution) before and after interactions with biological systems is simultaneously of particular interest and a substantial challenge [55]. Thus, correct characterization techniques should be used that match the intended use, with tailored checklists for specific nanoparticles. To overcome issues associated with any single technique, at least one other complementary characterization technique should be used [55]. The observed differences in the clinical profiles, the organ distribution and degradation kinetics of the different iron carbohydrate complexes suggest that the nanoparticle surface characteristics defined by the manufacturing process, and the different carbohydrates are the critical determinants of the drugs fate. It can be assumed that, upon intravenous injection, the particle surface will interact with its biological environment and govern differences in the uptake by the RES, leading to the observed distinct plasma clearance kinetics and tissue distribution and adverse events profiles. The critical structural surface attributes relevant for the interaction of the particles with the biological environment as well as their uptake and metabolism by the RES is not fully understood and requires further investigation.

## Figures and Tables

**Table 1:** Types of ligands used for parenteral iron preparations.

Ligand(s)	Trade Names	International Nonproprietary Names and/or Common Names	Ligand Description
Sucrose	Venofer^®^	Iron sucrose Iron saccharate Saccharated iron oxide	Disaccharide
	Fesin^®^		
	Ferrivenin^®^		
Dextran (polyisomaltose)	Imferon^®^ (HMWID)	Iron dextran	Polysaccharide, maltose units 1→6-linked
	Dexferrum^®^ (HMWID)		
	INFeD/Cosmofer^®^ (LMWID)		
Sorbitol and citric acid	Jectofer^®^	Iron sorbitex	Monosaccharide and carboxylic acid
Dextrin (polymaltose)	Amylofer^®^	Dextriferron Iron polymaltose	Polysaccharide, maltose units 1→4-linked
Gluconate and sucrose	Ferrlecit^®^	Sodium ferric gluconate	Carboxylic acid and disaccharide
Chondroitin sulfate	Blutal^®^	Iron chondroitin sulfate	Sulfated poly-glycosaminoglycan, alternating *N*-acetylgalactosamine and glucuronic acid
Carboxymaltose	Injectafer^®^/Ferinject^®^	Ferric carboxymaltose	Polysaccharide, maltose units 1→4-linked, oxidized
Polyglucose sorbitol carboxymethyl ether and mannitol (excipient)	Feraheme^®^	Ferumoxytol	Polysaccharide, maltose units 1→6-linked, hydrogenated and carboxymethylated, and monosaccharide
Isomaltoside 1000/derisomaltose, and citrate	Monoferric^®^/Monofer^®^	Iron isomaltoside 1000 Ferric derisomaltose	Oligosaccharide, maltose units 1→6-linked, hydrogenated, and carboxylic acid

HMWID: High-molecular-weight iron dextran; LMWID: Low-molecular-weight iron dextran.

**Table 2:** Composition of main iron carbohydrate preparations on the market (approximate values).

Product (Year of First Market Entry)	Carbohydrates	Iron Content (mg Fe/mL)	Carbohydrate Content (mg/mL)	Carbohydrate Content (mg/mg Fe)	pH
Iron sucrose [17] (1949) [27]	Sucrose	20	300	15	10.5–11.1
Sodium ferric Gluconate [18] (1951) [28]	Sucrose Gluconate	12.5	195 22	16 1.7	7.7–9.7
Iron dextran [19,20] (1974) [29]	Dextran	50	206	4.1	4.5–7.0
Ferric derisomaltose [22] (2009) [21]	Derisomaltose Citrate	100	230 10	2.3 0.1	5.0–7.0
Ferric carboxymaltose [24] (2007) [30]	Carboxymaltose	50	75	1.5	5.0–7.0
Ferumoxytol [26] (2009) [26]	polyglucose sorbitol carboxymethylether Maltose (excipient)	30	29 44	0.97 1.5	6–8

**Table 3:** Types of bonds present in the iron carbohydrate preparations.

Product	Carbohydrates	Van der Waals Forces	Non-ionic Hydrogen Bonds	Ionic Hydrogen Bonds	Coordinative Bonds
			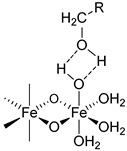	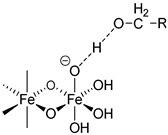	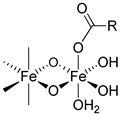
Iron sucrose	Sucrose	√	√	√	-
Sodium ferric gluconate	Sucrose Gluconate	√	√ √	√ -	- √
Iron dextran	Dextran	√	√	-	-
Ferric derisomaltose	Derisomaltose Citrate	√	√ √	- .	- √
Ferric carboxymaltose	Carboxymaltose	√	√	-	√
Ferumoxytol	polyglucose sorbitol carboxymethylether Maltose (excipient)	√	√ √	- -	√ -

R: residual of carbohydrate.

**Table 4:** Physico-chemical properties of iron carbohydrate complexes.

Product	Molecular Weight (kDa)	Particle Size (nm) [39]	Zeta Potential (mV) [39]	Crystalline Structure	Reduction Potential (mV) ° [25]	Reduction Kinetics k(Θ) × 10^3^ (min^−1^) Θ = 0.1/0.5/0.8 [25]	Blocking Temperature (K) [25,40]
Iron sucrose	34–60 [17,41] 42–44 [25] 252 [42] 140 [39]	8.3 (PDI 0.192)	pH 7.43: −26.20 pH 11.03 *: −28.15	2-line ferrihydrite [39] Ferrihydrite and lepidocrocite [41] Akageneite [33] 2-line ferrihydrite-like [40] No clear identification [25]	−494	107/89/117 ^§^	55
Sodium ferric gluconate	289–440 [18] 37.5 [9] 200 [42] 164 [39]	8.6 (PDI 0.244)	pH 7.4: −29.70 pH 8.36 *: −29.10	2-line ferrihydrite ([39] Ferrihydrite and lepidocrocite [41] Akaganeite [33]	nd	nd	nd
Iron dextran	165 [20] 165 [39]	12.2 (PDI 0.149)	pH 6.4 *: −15.30 pH 7.31: −17.25	Akageneite [39,41]	nd	nd	nd
Ferric derisomaltose	155 [22] 63–69 [25] 150 [39]	9.9 (PDI 0.182)	pH 6.3 *: −22.0 pH 7.35: −21.05	Akaganeite [25,39,41]	−338/−508	21/41/63	56
Ferric carboxymaltose	≈ 150 [24] 145–155 [25] 233 [39]	23.1 (PDI 0.07)	pH 5.36: 3.68 pH 7.26: −8.52	Akaganeite [25,39,41]	−333	18/35/55	114
Ferumoxytol	750 [26] 172–188 [25] 731 [42] 276 [39]	23.6 (PDI 0.143)	pH 6.6: −43.20 pH 7.36: −30.55	Magnetite/Maghemite [39] Magnetite [41] Maghemite [25]	−245/−768	36/67/98	73

*: Non-adjusted pH; °: two peaks, where applicable; mean of several batches, where applicable; §: Θ = 0.9 (Θ = 0.9 is 90% degradation); nd: not done; PDI: polydispersity index.

**Table 5:** Pharmacokinetic parameters of intravenous iron–carbohydrate complexes.

Parameter	Sodium Ferric Gluconate [9]	Iron Sucrose [9]	Ferric Carboxymaltose [9]	Ferumoxytol [9]	Ferric Derisomaltose [22]	Iron Dextran [19]
Dosage used for PK characteristics, mg Fe	125	100	100/1000	316	1000	NA
Terminal t_1/2_, h	1.42	5.3	7.4/9.4	14.7	27	20
C_max_, mg Fe/L	20.6	35.5	37/331	130	408	NA
AUC, mg Fe/L*h	43.7	83.3	333/6277	2912	17730	NA
AUC, standardized for a dose of 100 mg Fe, mg Fe/L*h	35.0	83.3	333/627	922	1773	NA
CL, L/h	2.99	1.23	0.26/0.16	0.11	NA	NA
IVIP-Fe * c_max_ (mmol/L) t_max_ (h) t1/2 (h) AUC_0-inf_ (h*mmol/L)		0.85 ± 0.17 0.35 (0.33–0.37) 3.43 ± 1.55 2.59 ± 0.5	1.16 ± 0.09 0.34 (0.33–0.7) 6.82 ± 1.93 12.39 ± 1.2		1.29 ± 0.1 0.67 (0.33–6) 20.3 ± 2.27 36.76 ± 4.9	

PK: pharmacokinetics; C_max_: maximum serum concentration; AUC: area under the curve; CL: clearance; IVIP: intravenous iron–carbohydrate complex preparation; t_max_: time to C_max_. * IVIP-Fe estimates drug bound iron in serum by subtracting transferrin bound iron from total serum iron [43]. All results are mean ± SD except for t_max_ (mean (min–max)). Dose administered: 200 mg Fe.

## Data Availability

Not applicable.
