# Correction: Funk et al. Criticality of Surface Characteristics of Intravenous Iron-Carbohydrate Nanoparticle Complexes: Implications for Pharmacokinetics and Pharmacodynamics. *Int. J. Mol. Sci.* 2022, *23*, 2140

**DOI:** 10.3390/ijms231810230

**Published:** 2022-09-06

**Authors:** Felix Funk, Beat Flühmann, Amy E. Barton

**Affiliations:** Vifor Pharma Management Ltd., Flughofstrasse 61, CH-8152 Glattbrugg, Switzerland

The authors wish to make the following corrections to this paper [1]:

In the original publication, there were wrong reference citations in paragraph 7 of Section 2.2 and Table 4 as published.

In Section 2.2, paragraph 7, “Ferumoxytol contains 30 mg of iron/mL. The complex is an iron oxide coated with polyglucose sorbitol carboxymethylether. The chemical formula of ferumoxytol is Fe_5874_O_8752_-C_11719_H_18682_O_9933_Na_414_ [24]” should be replaced with “Ferumoxytol contains 30 mg of iron/mL. The complex is an iron oxide coated with polyglucose sorbitol carboxymethylether [25]. The chemical formula of ferumoxytol is Fe_5874_O_8752_-C_11719_H_18682_O_9933_Na_414_ [26]”.

In Table 4, the reference numbers did not correspond with the correct numbers in the reference list. The corrected Table 4 appears below:
**Product****Molecular Weight (kDa)****Particle Size (nm)[39]****Zeta Potential (mV)[39]****Crystalline Structure****Reduction Potential (mV) °[25]****Reduction Kinetics k(Θ) × 10^3^ (min^−1^)** **Θ = 0.1/0.5/0.8[25]****Blocking Temperature (K) ** **[25,40]**Iron sucrose34–60 [17,41] 42–44 [25] 252 [42] 140 [39]8.3 (PDI 0.192)pH 7.43: −26.20 pH 11.03 *: −28.152-line ferrihydrite [39] Ferrihydrite and lepidocrocite [41] Akageneite [33] 2-line ferrihydrite-like [40] No clear identification [25]−494107/89/117 ^§^55Sodium ferric gluconate289–440 [18] 37.5 [9] 200 [42] 164 [39]8.6 (PDI 0.244)pH 7.4: −29.70 pH 8.36 *: −29.102-line ferrihydrite ([39] Ferrihydrite and lepidocrocite [41] Akaganeite [33]ndndndIron dextran165 [20] 165 [39]12.2 (PDI 0.149)pH 6.4 *: −15.30 pH 7.31: −17.25Akageneite [39,41]ndndndFerric derisomaltose155[22] 63–69 [25] 150 [39]9.9 (PDI 0.182)pH 6.3 *: −22.0 pH 7.35: −21.05Akaganeite [25,39,41]−338/−50821/41/6356Ferric carboxymaltose≈ 150 [24] 145–155 [25] 233 [39]23.1 (PDI 0.07)pH 5.36: 3.68 pH 7.26: −8.52Akaganeite [25,39,41]−33318/35/55114Ferumoxytol750 [26] 172–188 [25] 731 [42] 276 [39]23.6 (PDI 0.143)pH 6.6: −43.20 pH 7.36: −30.55Magnetite/Maghemite [39] Magnetite [41] Maghemite [25]−245/−76836/67/9873

The authors apologize for any inconvenience caused and state that the scientific conclusions are unaffected. This correction was approved by the Academic Editor. The original publication has also been updated.
